# Práticas médicas de redução de danos relacionados ao aborto inseguro na atenção primária à saúde na cidade de São Paulo, Brasil: contextos e barreiras para efetivação

**DOI:** 10.1590/0102-311XPT089124

**Published:** 2025-04-25

**Authors:** Nathália Machado Cardoso, Luiza Magalhães Cadioli, Mariana Pércia Namé de Souza Franco, Elisabeth Meloni Vieira, Ana Flávia Pires Lucas d’Oliveira

**Affiliations:** 1 Faculdade de Medicina, Universidade de São Paulo, São Paulo, Brasil.; 2 Faculdade de Saúde Pública, Universidade de São Paulo, São Paulo, Brasil.

**Keywords:** Aborto, Redução de Danos, Atenção Primária à Saúde, Medicina de Família e Comunidade, Abortion, Harm Reduction, Primary Health Care, Family Practice, Aborto, Reducción del Daño, Atención Primaria de Salud, Medicina Familiar y Comunitaria

## Abstract

O aborto induzido inseguro é uma questão de saúde pública responsável pela alta morbimortalidade no ciclo gravídico-puerperal. Diante da negligência do Estado para lidar com o problema, algumas estratégias de enfrentamento têm sido propostas. Uma delas é a atuação de profissionais de saúde na perspectiva de redução de danos relacionados aos abortos inseguros. Este estudo buscou identificar médicos e médicas da atenção primária à saúde que atuam nessa perspectiva, na cidade de São Paulo, Brasil, para compreender os desafios relacionados ao exercício desse cuidado. Trata-se de uma pesquisa qualitativa, de abordagem hermenêutico-dialética, por meio de entrevistas semiestruturadas com oito profissionais. Os resultados indicaram que essa atuação encontra muitos desafios, que vão desde o medo relacionado ao estigma e ao julgamento moral que permeiam o tema, passando pelo recrudescimento do conservadorismo no país e por entraves relacionados às políticas públicas de saúde, cujo alinhamento materno-infantilista norteia as práticas de cuidado no ciclo gravídico-puerperal. Como política pública direcionada, a estratégia de redução de danos precisa contar com vontade política, educação permanente, metas e indicadores para avançar na capacidade de responder às necessidades individuais de maneira integral e colaborar para a redução da mortalidade por aborto inseguro.

## Abstract (en)

Unsafe induced abortion is a public health problem responsible for high morbidity and mortality during the pregnancy and postpartum period. In view of the State’s negligence in addressing this problem, various preventive strategies have been proposed. One of them involves healthcare professionals working to reduce the risks of unsafe abortion. The aim of this study was to identify primary healthcare physicians in the city of São Paulo, Brazil, who embrace this perspective, and to understand the challenges they face in providing such care. It is a qualitative research with a hermeneutic-dialectical approach, conducting semi-structured interviews with eight professionals. The results show that this work faces many obstacles, including fear of stigma and moral judgement, the growth of conservatism in the country, and the limitations of public health policies, whose maternal and infant orientation guides care practices in the pregnancy-postpartum cycle. As a targeted public policy, harm reduction strategies require political commitment, continuous education, targets and metrics to comprehensively address individual needs and reduce mortality due to unsafe abortion.

## Abstract (es)

El aborto inducido inseguro es un problema de salud pública con alta morbimortalidad en el ciclo embarazo-puerperio. Ante la negligencia del Estado para hacer frente a este problema, se han propuesto algunas estrategias de afrontamiento. Una de ellas es la labor de los profesionales de la salud para reducir el daño de los abortos inseguros. Este estudio buscó identificar a los médicos de atención primaria de la ciudad de São Paulo (Brasil) que trabajan desde esta perspectiva, con el fin de comprender los desafíos relacionados con el ejercicio de esta atención. Se trata de una investigación cualitativa con enfoque hermenéutico-dialéctico en la que se aplicaron entrevistas semiestructuradas a ocho profesionales. Los resultados indicaron que el trabajo de los médicos está lleno de obstáculos, desde el miedo relacionado con el estigma y el juicio moral que conlleva el tema hasta la resurgencia del conservadurismo en el país y los obstáculos relacionados con las políticas de salud pública, cuya alineación materno-infantil guía las prácticas de atención en el ciclo del embarazo-puerperio. Como política pública específica, la estrategia de reducción de daños tendría que basarse en la voluntad política, en la educación permanente, en los objetivos y en los indicadores para responder a las necesidades individuales de manera integral y colaborar en la reducción de la mortalidad por aborto inseguro.

O aborto induzido é um procedimento simples quando realizado por profissionais capacitados, em condições sanitárias e com recursos adequados ^1^. Entretanto, dependendo do contexto legal, das condições socioeconômicas, do acesso e organização dos serviços de saúde e do estigma social, é possível que agreguem-se diferentes níveis de risco e insegurança ^2^.

O contexto brasileiro favorece a alta prevalência de abortos induzidos inseguros: a legislação é restritiva - determina que o aborto é crime, exceto nas gestações decorrentes de estupro, quando há risco de vida para a pessoa gestante e nos casos de anencefalia ^3^ ^,^ ^4^. A desigualdade social no Brasil - marcada por disparidades de gênero, raça, classe, entre outras - é uma das maiores do mundo ^5^, o que influencia no acesso à informação e aos serviços de saúde reprodutiva ^6^, permeado por incontáveis barreiras ^7^, e o estigma social em torno do tema é bastante elevado ^8^. Por essa razão, embora saibamos que nem todo aborto induzido ilegalmente é inseguro ^2^, e que nem todo aborto legal é seguro ^2^, adotamos a terminologia aborto inseguro para os casos de abortos não previstos em lei, tema abordado neste artigo.

A gestação imprevista é um evento no qual se entrelaçam processos decisórios em níveis individuais, contextuais e relacionais ^9^. Quando ocorre fora das condições legais para abortamento, as alternativas incluem continuidade da gestação ou realização de aborto inseguro. Esse dilema é particularmente prevalente no Brasil, onde 55,4% ^10^ das gravidezes são imprevistas e, aos 40 anos, uma em cada sete mulheres já interrompeu uma gestação ^11^. Estudos indicam uma magnitude anual de até quase um milhão de casos, com significativa morbimortalidade associada ^12^ ^,^ ^13^.

Diante do quadro de injustiça reprodutiva no país, movimentos sociais e profissionais buscam estratégias para mitigar danos fora e dentro do sistema de saúde: no primeiro grupo, ativistas feministas oferecem apoio na autogestão de abortos ^14^; no segundo, profissionais de saúde realizam consultas de aconselhamento a pessoas passando por gestação imprevista em uma atuação que, a depender do contexto, tem potencial de reduzir significativamente a morbimortalidade por aborto inseguro ^15^ ^,^ ^16^, mais conhecida como estratégia redução de danos.

Para evitar ambiguidades linguísticas e reconhecer o poder das ideias e práticas vindas dos movimentos sociais ^17^, ressaltamos que embora o termo “redução de danos” apareça na literatura - e no presente artigo - como uma estratégia de saúde pública baseada em evidências que busca reduzir os danos de atividades específicas, em vez de proibi-las ^18^, sua origem remonta à atuação de ativistas de movimentos sociais para descrever práticas de ação direta e de rua com usuários de drogas ilícitas em contextos comunitários independentes do controle estatal e institucional, ancoradas em justiça social e direitos humanos ^17^. Com o passar do tempo tal termo foi adaptado e utilizado em diversos contextos, incluindo o de profissionais de saúde que aconselham pessoas que necessitam abortar mas vivem em condições altamente restritivas ^17^, objeto deste estudo. Aqui utilizaremos redução de danos para nos referirmos à atuação de profissionais de saúde em contexto de cuidado a pessoas em situação de risco relacionado à prática de abortos inseguros, tendo como referência principal o modelo pioneiro desenvolvido no Uruguai em momento anterior à legalização, em 2012, quando o aborto inseguro era a principal causa de morte materna no país ^19^.

A implementação de consultas de aconselhamento em redução de danos visa garantir que, diante da suspeita de gestação imprevista, os profissionais de saúde estejam capacitados para ^19^:

(1) Confirmar a gestação imprevista;

(2) Oferecer escuta e acolhimento sem julgamentos, identificando a decisão da pessoa sobre a gestação;

(3) Identificar casos elegíveis para aborto legal, informar sobre o direito e realizar os encaminhamentos necessários;

(4) Fornecer informações sobre os riscos do aborto clandestino, considerando a idade gestacional e os métodos comumente utilizados;

(5) Explicar os métodos mais seguros de aborto;

(6) Programar consulta de retorno para iniciar o pré-natal ou cuidados pós-aborto, conforme a decisão da pessoa atendida.

No Brasil, a norma técnica de atenção humanizada ao abortamento (2005) ^20^ orienta quanto à importância de se prestar assistência humanizada, seguindo princípios éticos, legais e bioéticos que promovam acolhimento e atenção às necessidades biopsicossociais das pessoas gestantes de modo bastante semelhante ao previsto na atuação em redução de danos - atitudes que deveriam ser realizadas por qualquer profissional de saúde, em qualquer nível de atenção. No entanto, a política uruguaia progrediu na implementação do direito de acesso à informação sobre o avanço científico e suas respectivas aplicações ^21^ ao incluir o fornecimento de orientações acerca de métodos comumente usados e mais seguros de abortamento, como o misoprostol ^12^ ^,^ ^22^.

Assim, a atuação em redução de danos reforça a autodeterminação reprodutiva como um direito ^23^, possibilitando que abortos de alto risco se tornem menos inseguros ^24^, mesmo quando não previstos em lei. Para isso, a pessoa que gesta precisa ser reconhecida, no encontro assistencial, como um sujeito com saber próprio e capacidade decisória.

Essa perspectiva dialoga com a noção de cuidado como parte do plano do fazer da integralidade, tal como definido por Ayres ^25^. Para o autor, cuidado pressupõe o encontro intersubjetivo entre pessoas que buscam os serviços e os profissionais de saúde, e envolve vínculo e responsabilidade para uma potencial expressão de intersubjetividades ricas e plurais ^26^. Tais subjetividades estão diretamente relacionadas às condições macropolíticas, da organização dos serviços e das relações interpessoais estabelecidas nos espaços assistenciais ^25^.

Em termos do local de produção do cuidado, no contexto brasileiro, as unidades básicas de saúde (UBS) constituem-se como estratégia-chave para a redução de danos relacionados a abortos inseguros ^16^ ^,^ ^27^. Isso porque a atenção primária à saúde (APS) é o primeiro ponto de contato para os problemas de saúde mais comuns da população e é responsável pela resolução da maioria deles ^28^ ^,^ ^29^. É nas UBS que as equipes acolhem a população residente no território, seguindo longitudinalmente os indivíduos e as famílias ^28^. Parte importante dos serviços tem Estratégia Saúde da Família (ESF) e conta com médicos de família e comunidade ^28^.

No entanto, não há um protocolo do Ministério da Saúde que oriente as equipes das UBS para redução de danos, mas existe uma cartilha elaborada pelo Grupo de Trabalho Mulheres na Medicina de Família e Comunidade da Sociedade Brasileira da Medicina de Família e Comunidade (SBMFC) em parceira com a Anis - Instituto de Bioética ^30^, publicada em 2022 e disseminada entre os profissionais dessa especialidade, com orientações para redução de danos.

Uma pesquisa nas bases de dados SciELO, BVS e Google Acadêmico realizada pelas autoras não identificou artigos que descrevam as práticas assistenciais de redução de danos a abortos inseguros no Brasil. Reconhecendo essa lacuna de conhecimento, buscamos identificar médicos que atuam na perspectiva de redução de danos em relação ao aborto inseguro na APS em São Paulo, a fim de caracterizar e compreender as ações realizadas sob a perspectiva do cuidado, identificando desafios, lacunas e potencialidades.

## Método

Os dados empíricos que apoiam a discussão apresentada fazem parte de uma pesquisa mais ampla sobre aborto, redução de danos e cuidado.

A produção dos dados norteou-se pela abordagem qualitativa, utilizando a técnica de entrevista semiestruturada com roteiro ^31^ sobre os temas: motivações e sentimentos mobilizados, ações realizadas e entraves encontrados para o exercício do cuidado em redução de danos.

Convidamos a participar da pesquisa médicos da APS no Município de São Paulo, que trabalhassem em equipes de ESF no Sistema Único de Saúde (SUS) e afirmassem realizar atendimentos na perspectiva da redução de danos ao aborto inseguro, acolhendo gestações imprevistas. A partir dos primeiros contatos, seguimos a estratégia de bola de neve ^31^.

Os participantes foram recrutados em duas frentes distintas: (i) convite para participação enviado por mensagem de texto em um grupo de WhatsApp composto por 180 médicos de família e comunidade de todo o Brasil; e (ii) contato direto com um profissional, médico de família e comunidade, próximo à primeira autora do artigo, que ralizou todas as entrevistas.

Foram realizadas oito entrevistas entre outubro de 2023 e 2024, após o esclarecimento e a assinatura do Termo de Consentimento Livre e Esclarecido. Os locais de realização das entrevistas foram de escolha dos entrevistados, com garantia de privacidade. Todas as entrevistas foram realizadas pela mesma pesquisadora, gravadas em áudio e transcritas na íntegra para análise. As transcrições foram realizadas por pessoa com contrato explícito de sigilo e confidencialidade. O número final de entrevistados foi definido usando o critério de saturação ^32^ de falas e significados atribuídos à experiência.

O processo analítico-interpretativo seguiu o método hermenêutico-dialético ^31^. Como categorias analíticas, utilizamos a perspectiva dos direitos reprodutivos, da redução de danos e do cuidado em saúde enquanto prática intersubjetiva. Partimos do pressuposto de que a clínica, enquanto prática social relacional, articula a racionalidade técnica com um sistema de valores e de diretrizes ético-políticas que exige capacidade reflexiva para a tomada de decisão compartilhada durante o processo de trabalho ^33^. Assim, discutimos as potencialidades e os limites, os entraves e as barreiras que surgem no fazer prático de profissionais que atuam na perspectiva da redução de danos.

Como categorias empíricas e operacionais, definimos três: elementos de redução de danos presentes ou não na consulta; medo de represálias e/ou estigma em torno do tema do aborto; e dificuldades relacionadas à organização dos serviços e às diretrizes das políticas públicas.

A análise final buscou apresentar respostas para esclarecer a lógica interna do grupo sobre o tema, e a síntese interpretativa foi articulada com a literatura e com a fundamentação teórica inicial.

As autoras do artigo são médicas, duas delas médicas de família e comunidade, uma ginecologista e duas sanitaristas, e atuam ou atuaram na perspectiva dos direitos reprodutivos e da redução de danos a abortos inseguros. Esta característica, ao mesmo tempo que as faz compreender vários dos desafios colocados com facilidade, desafia a estranhar o familiar ^34^, já que o familiar não é necessariamente conhecido. Na análise, mantiveram postura crítica e de estranhamento daquilo que é familiar, para desvelar os desafios e a diversidade dos profissionais entrevistados.

A pesquisa foi registrada na Plataforma Brasil e aprovada pelo Comitê de Ética e Pesquisa do Hospital das Clínicas da Faculdade de Medicina da Universidade de São Paulo (parecer nº 6.314.710).

## Resultados e discussão

Os resultados visam analisar a atuação dos profissionais na redução de danos de abortos fora da legalidade. Embora a identificação de permissivos legais e as condutas necessárias a esses casos sejam parte da redução de danos, tais competências não foram incluídas neste artigo.

As características dos profissionais entrevistados, a duração das entrevistas e os materiais usados como norteadores das práticas relatadaQWs estão descritos no Quadro 1. Seus nomes foram substituídos pela letra “P”, acompanhada de um número, para preservar identidades.

**Quadro 1:** Características dos entrevistados, tempo de duração de entrevistas e materiais usados como norteadores das práticas.

ENTREVISTADO	IDADE (ANOS)	GÊNERO	RAÇA/COR	DURAÇÃO DA ENTREVISTA (MINUTOS)	REGIÃO DE ATUAÇÃO	FORMAÇÃO	MATERIAL CITADO COMO NORTEADOR DA PRÁTICA
P1	36	Feminino	Branca	46	Sul	Residência em Medicina de Família e Comunidade	Cartilha da SBMFC (2022) ^30^
P2	32	Masculino	Branca	53	Oeste	Residência em Medicina de Família e Comunidade	*Abortamento Seguro: Orientação Técnica e de Políticas para Sistemas de Saúde* (2013) ^60^; site Women Help Women (https://womenhelp.org/); site Como Fazer Aborto Seguro (https://comofazerabortoseguro.org/); guia do teste rápido de gestação do Ministério da Saúde (2013) ^39^
P3	31	Feminino	Branca	55	Sul	Residência em Medicina de Família e Comunidade	*Abortamento Seguro: Orientação Técnica e de Políticas para Sistemas de Saúde* (2013) ^60^
P4	31	Masculino	Branca	40	Sul	Residência em Medicina de Família e Comunidade	Cartilha da SBMFC (2022) ^30^
P5	30	Feminino	Branca	48	Oeste	Residência em Medicina de Família e Comunidade, pós-graduanda em Saúde Coletiva	Cartilha da SBMFC (2022) ^30^
P6	29	Feminino	Branca	65	Sul	Residência em Medicina de Família e Comunidade	Cartilha da SBMFC (2022) ^30^
P7	31	Feminino	Branca	73	Sudeste	Residência em Medicina de Família e Comunidade	Cartilha da SBMFC (2022) ^30^
P8	41	Feminino	Negra	38	Oeste	Título em Medicina de Família e Comunidade	Norma técnica de atenção humanizada ao abortamento (2011) ^61^; bula do misoprostol; *Abortion Care Guideline* (2022) ^47^; guia do teste rápido de gestação do Ministério da Saúde (2013) ^39^

SBMFC: Sociedade Brasileira de Medicina da Família e Comunidade.

Apenas dois dos entrevistados relataram contato com o tema do cuidado em redução de danos para abortamento inseguro durante o período da graduação, em atividades extracurriculares. Eles deixaram claro que o tema do aborto é pouco explorado na faculdade de Medicina, de maneira que, ao se formarem, não se sentiram capacitados para lidar com situações de gestação imprevista que não se enquadram nos permissivos legais. Sete deles relataram que a especialização na residência foi um momento determinante para esse aprendizado. Esse achado corrobora a literatura sobre a formação médica, que aponta que a abordagem do tema na graduação é influenciada pela política institucional, focada apenas no âmbito biológico e legal ^35^. A residência médica, especialmente para os médicos de família e comunidade, é um momento importante para discutir gênero, sexualidade e desenvolver habilidades de comunicação e acolhimento ^35^. Isso ocorre, no entanto, apesar de o Currículo Baseado em Competências da SBMFC não enfatizar o manejo de situações de gestação imprevista e risco de aborto inseguro como habilidade essencial ^27^.

Como motivação para atuação, cinco das seis médicas declararam-se feministas. No conjunto dos oito entrevistados, cinco relacionam a atividade à garantia dos direitos humanos e à justiça social ^36^. Outra justificativa relevante refere-se à alta prevalência do aborto inseguro, uma vez que agravos com elevada prevalência são de competência da APS ^28^, conforme indicado por três informantes.

“*Então, eu sou declaradamente feminista, eu acho que estar na atenção primária é lidar com esse tipo de situação. É um problema de saúde pública e eu acho que, além de tudo isso aí, não é meu direito regular a vida dos outros*” (P8).

O desejo de ser um(a) profissional da saúde capacitado, apoiar quem passa pelo problema e facilitar o acesso à informação surgiu em quatro discursos, como um contraponto aos colegas médicos, que consideram ser um obstáculo para bons cuidados, fato já apontado na literatura ^37^. Por último, o risco do aborto inseguro é lembrado por três entrevistadas, que citam como motivação evitar mortes e sequelas para realizar o seu trabalho a contento.

### Elementos de redução de danos presentes, ou não, na consulta

Todos os médicos de família e comunidade afirmaram questionar o planejamento e desejo da gestação no primeiro atendimento, independentemente de a pergunta já ter sido feita e registrada em prontuário por outro profissional.

“*Eu tenho a postura de sempre perguntar. Então, no primeiro atendimento daquela gestante, que normalmente não é o primeiro atendimento de gestação dela, porque normalmente já passou com a enfermeira quando chega comigo, eu sempre pergunto se aquela foi uma gestação planejada. Aí, se ela fala que não, eu pergunto: ‘Mas como é que você tá se sentindo sobre isso?’*” (P8).

Todos os entrevistados disseram valorizar o uso de habilidades de comunicação, como o método clínico centrado na pessoa ^38^, e a realização de orientação pré e pós-teste rápido de gestação, com menção de dois deles ao guia técnico de teste rápido de gravidez (TRG) na atenção básica ^39^, do Ministério da Saúde, como um bom roteiro para nortear a abordagem de desejos e expectativas diante da gravidez.

O guia, lançado em 2013 como parte das ações da Rede Cegonha ^40^, define a oferta de TRG “*não apenas como insumo, mas como dispositivo que oportuniza o diálogo sobre a saúde sexual e a saúde reprodutiva*” ^39^ (p. 5). Em suas orientações, indica que os profissionais devem acolher “*os diversos significados que a reprodução pode ter para cada pessoa, em diferentes momentos da vida*” ^39^ (p. 5).

No entanto, embora tais orientações estejam contidas em algumas diretrizes ^20^ ^,^ ^41^, o que se revela é que, ainda que o profissional esteja convicto da importância de abordar o assunto da gestação imprevista, esse não é um tema fácil de introduzir no encontro clínico. Alguns profissionais consideraram precisar conhecer os valores da pessoa gestante antes de falar sobre o tema, e isso inclui uma comunicação que nem sempre é fluida.

“*Não é tão fluido assim, então tem uma coisa de ser meio truncado o começo da conversa, até por não saber direito quem é essa pessoa que você tá falando, o que ela tá pensando... mas talvez você só saiba se perguntar, né?!*” (P2).

Empenhados em realizar uma escuta solidária e sensível aos sofrimentos, os médicos frequentemente se deparam com questionamentos como: quem é, de fato, meu interlocutor? Quais são suas opiniões sobre o tema do aborto? Da mesma forma, os pacientes podem refletir sobre as crenças e atitudes dos profissionais. Esse processo evidencia como as dimensões do sujeito e da intersubjetividade influenciam as práticas de saúde e ressalta a importância do vínculo para o (re)conhecimento e expressão de intersubjetividades capazes de produzir o cuidado ^26^ ^,^ ^42^. Sem vínculo de confiança, perde-se a oportunidade de atuação positiva com a alteridade revelada no desencontro ^33^.

Uma das entrevistadas ilustra bem esse interesse em promover o diálogo ^26^:

“*Sempre que eu vejo que tem algum contexto de que talvez não era o que ela queria, a questão social, o marido, a religião que não permite, eu tento mostrar que eu não sou essas... não sou esses limitantes, entende?! E aí vejo se ela também quer falar sobre*” (P7).

A dificuldade dos profissionais em abordar gestantes nos 2º e 3º trimestres é maior. Embora todos investiguem a gestação imprevista, alguns perceberam limitações nas práticas e refletiram sobre perguntar menos a respeito das perspectivas das pessoas atendidas em gestações mais avançadas:

“*Eu pergunto menos, pergunto menos. Geralmente pergunto: ‘E essa gravidez, foi planejada?’ Muitas vezes não foi, mas ‘como tá agora?’ E muita gente fala: ‘A cabeça ficou a milhão, mas agora tá tudo bem.’ E daí, eu não continuo muito. Eu sinto que eu pergunto menos ou aceito mais rápido as explicações. Parece mais difícil, parece que já tá estabelecido, entende?!*” (P2).

Esses profissionais demonstram sentimento de impotência para lidar com os casos, com sensação de que ficam de “mãos atadas”, pois acreditam que não há o que possa ser feito. Como o início tardio e a baixa adesão ao pré-natal são comuns em gestações imprevistas ^43^, algo também percebido pelos profissionais, a dificuldade de abordar o tema resulta na perda de oportunidade para identificar pessoas em risco de buscar procedimentos inseguros.

Isso pode estar relacionado ao fato de que a Organização Mundial da Saúde (OMS) ^44^ recomenda a autogestão segura do aborto, sem o auxílio de profissionais de saúde até 12 semanas de gestação. Não podemos ignorar que a prática médica é socialmente ofertada e demandada como intervenção segura ^45^, baseada em evidências cientificas ^46^. Isso explica certo tensionamento da ação em redução de danos nesses casos, situações em que os médicos adotam postura mais pragmática, pois compreendem que nada pode ser feito para evitar um aborto inseguro. Diante da impotência terapêutica, eles se percebem limitados em suas atuações e perdem a dimensão do que é possível fazer, evitando danos, acolhendo sofrimentos e comunicando direitos.

Por fim, pode ser que o avanço da idade gestacional traga um tensionamento maior, relacionado à moral, que dificulta conversar sobre o tema.

Uma das entrevistadas defendeu reconhecer a gravidez indesejada mesmo com idade gestacional avançada, neste sentido:

“*Até pra entender com o que que eu vou lidar naquele pré natal. Porque mesmo que ela vá levar a gravidez até o final, a gravidez pode trazer bastante sofrimento e a gente dar espaço para as pessoas falarem sobre esse sofrimento gerado pela gravidez é importante*” (P8).

Em relação às orientações, todos os profissionais abordaram os riscos envolvidos no uso de técnicas inseguras e explicaram sobre métodos seguros, nomeando o misoprostol, que é recomendado pela OMS ^47^. Embora nenhum tenha se recordado das posologias de uso, sete deles relataram consultar sites de confiança para indicar dose e vias de administração, e uma afirmou que não faz esse tipo de orientação. Esse fato é importante, uma vez que a literatura aponta uma lacuna em relação à informações sobre a forma correta de proceder com o aborto - sendo essa a agenda principal das pessoas gestantes, bem como a busca pela medicação ^22^ -, e que a reconhecida eficácia do medicamento muitas vezes se desestabiliza por equívocos cometidos devido à sua situação de substância ilícita no país ^48^. Por fim, há um reconhecimento geral por parte dos entrevistados de que a impossibilidade de acesso legal ao misoprostol é um entrave para a atuação em redução de danos.

Sete dos oito médicos de família e comunidade mencionaram ser comum a falta das pessoas na consulta de retorno, exceto quando decidem seguir com o pré-natal. Somente duas profissionais relataram fazer busca ativa para elucidar o motivo das faltas. Isso pode ser repercussão da noção da clandestinidade e da ilegalidade em torno do tema, enquanto nem profissional de saúde e nem paciente retomem o assunto do aborto, ainda que ele já tenha sido amplamente discutido entre os dois. O que parece é que, quanto menos se fala no assunto, melhor.

“*O caso típico é fazer esse primeiro acolhimento e aí essa mulher some, desaparece...*” (P5).

A ausência da consulta de seguimento e/ou da vigilância dos casos e a dificuldade de conversar sobre o assunto chamou atenção, uma vez que a longitudinalidade e a coordenação de cuidado, como atributos ^29^, e a assistência programada ao pré-natal ^49^ são princípios que pautam a atuação dos profissionais da APS. Assim, perde-se oportunidade para a realização de cuidados clínicos imediatos, acolhimento às necessidades dos indivíduos e oferta de planejamento reprodutivo.

A abordagem em redução de danos, na prática cotidiana, revelou desafios, trazidos pelos relatos dos médicos de família e comunidade. Desvelam-se dificuldades para incluir o risco de interrupção insegura da gravidez no escopo assistencial, independentemente das convicções ético-políticas do trabalho. Mesmo cientes sobre o que é correto, norteados por princípios éticos e de direitos humanos e utilizando materiais diversos para nortear o trabalho, os profissionais atuam em redução de danos de forma quase clandestina, o que compromete o vínculo e está intimamente relacionado a um elemento que atravessou todas as entrevistas: o medo.

### Medo de represálias e/ou estigma em torno do tema do aborto

Nenhum entrevistado acreditou atuar fora da lei, mas todos mencionaram a influência da ilegalidade do aborto, da moral e do estigma em suas ações, gerando receio. Por entenderem que uma parcela da sociedade considera o aborto como imoral, eles destacaram que temem sofrer punições do órgão de classe, críticas de colegas, pacientes e familiares, ou estigmatização. Alguns avançam mais, outros menos, nos cuidados, dependendo da relação com pacientes e com a equipe na qual trabalham.

Alguns profissionais mencionaram o medo de orientar sobre redução de danos e serem denunciados pela própria gestante. Ou seja, ao mesmo tempo que querem estabelecer vínculo e confiança, há receio de abordar o assunto:

“*E eu sempre tenho uma questão também de proteção minha, porque eu não vou falar abertamente em uma situação que eu vejo que o meu território tem uma quantidade maior de pessoas, que, desculpa, têm uma tendência a serem contra o aborto*” (P1).

Sete profissionais compartilharam a preocupação de ocultar a atuação em redução de danos dos colegas, devido à ilegalidade do tema, ao fortalecimento da extrema-direita ^50^ e à influência de ambos no contexto do trabalho regulado por um conselho de classe conservador ^51^. Ou seja, esses médicos temem reações negativas, como boatos, o rótulo de “aborteira” ou até denúncias ao conselho.

“*O medo vem dos pares, de não compreenderem. Porque, apesar de não ser ilegal, tem o lugar da imoralidade, pra dentro da equipe*” (P6).

“*É mais relacionado a questões judiciais mesmo, questões jurídicas de me processarem e eu ser, sei lá, punida de algum jeito por isso e perder o meu CRM, por exemplo*” (P1).

O medo dos profissionais é fundamentado nas recentes medidas do Conselho Federal de Medicina (CFM) ^52^ e do Conselho Regional Medicina do Estado de São Paulo (CREMESP) ^53^ contra médicas de serviços de aborto legal. Tais ações, embora não sejam relacionadas à atuação em redução de danos, cumprem o papel de deixar os profissionais temerosos em acolher e orientar. Vale lembrar que, apesar de suas ações estarem circunscritas aos limites éticos da profissão ^54^, a prática de redução de danos encontra-se em disputa em relação à sua institucionalidade.

Os profissionais se sentem mais seguros em compartilhar o trabalho quando conhecem a postura política do colega e percebem uma tendência menos conservadora ou religiosa. A relação entre os pares impacta diretamente o cuidado, tornando-o mais ou menos multidisciplinar, a depender desse alinhamento. Tal fato reforça como o vínculo também entre os membros da equipe afeta diretamente o cuidado no espaço assistencial ^25^.

“*...Eu não me sinto confiante quando eu falo: ‘Ah, vou falar em uma reunião’. Eu não sei se eu posso, entendeu?*” (P3).

Uma entrevistada relatou a influência da presença de uma colega durante um atendimento, situação que fez com que ela deixasse de orientar a posologia de uso do misoprostol, avançando na atuação em redução de danos:

“*Eu fui ficando com mais medo, principalmente perto de outros profissionais. Então, quando tô em atendimento compartilhado lá na UBS, eu não chego a falar de dose. Porque eu tenho medo. Eu prefiro falar com a mulher sozinha*” (P6).

Assim, o que vemos é uma atuação essencialmente solitária, cuja articulação entre os membros das equipes é determinada pelas ideias que os sujeitos têm sobre as posições políticas, religiosas e opiniões dos colegas em relação ao tema do aborto.

### Dificuldades relacionadas à organização dos serviços e às diretrizes das políticas públicas

Dois elementos centrais foram apontados como entraves na organização do trabalho: a pressão pela abertura do SisPrenatal e a identificação tardia dos casos.

Os médicos de família e comunidade relataram fluxos semelhantes nas unidades, em que pessoas que confirmam a gestação são priorizadas e atendidas pela enfermagem, que realiza a conversa inicial e insere os dados no SisPrenatal ^55^ - o que, na visão dos profissionais, é feito sem que se leve em conta os sentimentos da pessoa atendida diante da revelação da gestação.

“*Acho que se a paciente meio que falar abertamente, sei lá, começa a chorar, aí talvez eles chamem algum dos médicos pra conversar. Mas se for depender de alguém perguntar ativamente, tentar entender a situação, não sei o quanto que, no fluxo atual da unidade, vai rolar*” (P2).

O fluxo fechado e bem estabelecido nas unidades de saúde é reflexo de metas e indicadores utilizados para avaliar a boa assistência ao pré-natal, repassar verbas e monitorar o desempenho das UBS. No entanto, esse fluxo praticamente interdita a precoce identificação da gravidez imprevista e o cuidado dos casos, dificultando a expressão dos sentimentos e decisões das pessoas gestantes e prejudicando cuidado:

“*...O pré-natal é uma meta muito importante, entrou como meta agora a abertura precoce do pré-natal. Se a gente tem muitas gestantes abrindo depois de 12 semanas, a Organização Social de Saúde que gerencia a região é penalizada, então tem meio que uma pressa*” (P2).

A necessidade de cumprir metas foi evidenciada por uma entrevistada, que destacou a dificuldade em dialogar com a enfermeira de sua equipe a respeito de seu entendimento de que elas não deveriam conduzir a abertura do SisPrenatal em um caso que atenderam juntas:

“*A princípio, a enfermeira da minha equipe falou que teria que abrir. E aí eu falei pra ela: ‘Vamos conversar, então, com a RT* [responsável técnica]*’. E ela ficou com muito medo de conversar com a RT, essa coisa da hierarquia da enfermagem. Tem muito medo dentre a equipe de enfermagem de não abrir, de irem cobrar depois, principalmente agora com essa cobrança dos indicadores das gestantes*” (P6).

Todos os profissionais afirmaram adiar ou não abrir o SisPrenatal, desafiando o fluxo imposto e, às vezes, pactuando esse acordo com as gestantes. Essa diferença de conduta em relação à enfermagem pode refletir a desigualdade de poder entre os trabalhadores, com maior autoridade do médico ^56^.

A abertura precoce do SisPrenatal prejudica a identificação dos casos pelos médicos de família e comunidade, pois adia a consulta para o retorno com os exames. Como a próxima consulta é agendada para um mês após a primeira visita, o cuidado em redução de danos é postergado. Como já vimos, o avanço da gestação dificulta a atuação em redução de danos dos médicos, ressaltando a importância do envolvimento de profissionais de enfermagem nessa atuação.

“*Normalmente quando chega comigo, já passou, já abriu o pré-natal, já fez o diagnóstico, já fez exame, já fez muita coisa. Então, acho que a gente perdeu muitos momentos*” (P5).

Fato é que os protocolos que norteiam as práticas da APS ^49^ ^,^ ^57^ ^,^ ^58^ abordam apenas superficialmente o tema da gestação imprevista, limitando-se a ações preventivas ligadas à contracepção. Assim, há um duplo silenciamento sobre o papel da APS no cuidado de situações de risco para aborto inseguro, tanto nas diretrizes sobre interrupção da gestação ^27^ quanto nos instrumentos que orientam as práticas assistenciais nesse nível de atenção.

As metas mencionadas pelos profissionais refletem a Rede Cegonha ^40^ e evidenciam os entraves relatados no nível da gestão. Nela, encontramos diversas orientações sobre vigilância e cuidado no pré-natal, sem qualquer indicador relacionado a casos de risco de aborto inseguro. A Rede Cegonha é uma estratégia do Ministério da Saúde implementada em meio à forte oposição dos movimentos de mulheres e de saúde ^59^, porque representou um retrocesso em relação a conquistas prévias que ampliaram o escopo da saúde das mulheres para além da reprodução, o chamado “materno-infantilismo” ^59^.

Com metas e indicadores atrelados apenas à captação e continuidade do pré-natal, as diretrizes falham ao não fornecerem orientação técnica em relação aos cuidados de prevenção ao aborto inseguro. Assim sendo, a APS, que deveria ser a porta de entrada para a assistência integral, apresenta-se como mais uma barreira para o efetivo cuidado.

## Conclusão

Embora não seja uma política pública oficial, a estratégia de redução de danos no enfrentamento do aborto inseguro tem sido adotada por alguns profissionais da APS, essencialmente os médicos de família e comunidade. É importante reconhecer o empenho desses profissionais, cujas atuações refletem um alinhamento ético-político do trabalho, pautado por valores de justiça reprodutiva, reconhecendo as pessoas que gestam como sujeitos de direitos, com grande esforço individual para oferecer acolhimento, escuta sem julgamentos e informações em um contexto complexo. A existência desses profissionais nos traz a possibilidade de disseminação de práticas que poderiam salvar vidas e reduzir sofrimento mesmo sem mudanças na legislação.

A atuação em redução de danos revelou-se heterogênea entre os médicos de família e comunidade, sendo fortemente influenciada pelo contexto institucional e pelas concepções que eles têm sobre os valores morais e as posições políticas e religiosas dos membros das equipes de saúde e das pessoas atendidas. Quando há o temor de represálias e estigmatização, o vínculo de confiança é enfraquecido, tanto entre médicos de família e comunidade e pessoa atendida, quanto entre médicos de família e comunidade e demais membros das equipes de saúde. Essa realidade faz com que o trabalho em redução de danos seja percebido como uma ação essencialmente solitária e arriscada, a despeito da convicção pessoal em relação à sua legalidade. Assim, a implementação dessa prática precisa de uma abordagem mais sistematizada e ampla para garantir sua eficácia em nível populacional.

Entraves identificados para o trabalho estão relacionados ao recrudescimento do conservadorismo, que contribui para o clima de insegurança dos profissionais, e às diretrizes materno-infantilistas presentes nos documentos que orientam as práticas de saúde no cuidado durante o ciclo gravídico-puerperal. Para lidar com esses desafios é fundamental o investimento em educação continuada nos serviços de saúde, favorecendo a discussão de todos os membros da equipe sobre como ouvir e responder ao desejo de interrupção de gravidez, reduzindo riscos dentro do marco legal. Ser capaz de abordar o tema independentemente de religião ou convicções morais, mas a partir da saúde, seria importante para uma política que não dependesse apenas dos valores de cada trabalhador.

Também é de grande importância o investimento no ensino de comunicação clínica durante a graduação e especialização nas áreas da saúde, passo fundamental para promover um cuidado mais humano e eficaz, capacitando estudantes para lidarem com as complexidades emocionais e sociais envolvidas nesse contexto. Para isso, é necessário incluir na grade curricular a discussão sobre autodeterminação reprodutiva, moral e estigma, escuta, acolhimento sem julgamentos e princípios da redução de danos ao aborto inseguro.

Por fim, a incorporação da estratégia de redução de danos sem um compromisso ético da gestão é muito difícil. Assim, a implementação de protocolos claros e indicadores de saúde atrelados à identificação de gestação imprevista e mitigação de risco de aborto inseguro poderia melhorar a capacidade da APS em responder às necessidades individuais das pessoas em um momento crucial de sua saúde reprodutiva e colaborar para a redução da mortalidade relacionada ao ciclo gravídico puerperal.

O estudo foi realizado em um município onde há perseguição a médicos, mas também sugere que, embora o medo e o estigma sejam questões amplamente discutidas em nível nacional, as realidades podem variar em diferentes regiões. Outra questão é que os participantes foram escolhidos por praticarem redução de danos de aborto inseguro. Provavelmente médicos da APS sem especialização tenham perspectivas e ações bem diferentes, sendo importante conhecê-las. Também é fundamental compreender as concepções e práticas de redução de danos adotadas por profissionais de enfermagem na APS, considerando o papel essencial que desempenham nas equipes de ESF. Devido à relação estreita com equipe e comunidade, estudos sobre as concepções e práticas de agentes comunitários de saúde em torno do tema podem oferecer perspectivas valiosas que contribuam para a ampliação da abordagem de redução de danos de abortos inseguros. De igual importância - ainda que mais desafiadoras - são as pesquisas que analisem os desfechos da atuação em redução de danos e a perspectiva das pessoas atendidas.
